# How unmeasured confounding in a competing risks setting can affect treatment effect estimates in observational studies

**DOI:** 10.1186/s12874-019-0808-7

**Published:** 2019-07-31

**Authors:** Michael Andrew Barrowman, Niels Peek, Mark Lambie, Glen Philip Martin, Matthew Sperrin

**Affiliations:** 1 University of Manchester, Vaughan House, Portsmouth Street, Manchester, M13 9GB UK; 2 Institute for Science and Technology in Medicine, Keele University, Stoke-on-Trent, ST4 7QB UK

**Keywords:** Competing risks, Unmeasured confounding, Simulation study, Observation studies

## Abstract

**Background:**

Analysis of competing risks is commonly achieved through a cause specific or a subdistribution framework using Cox or Fine & Gray models, respectively. The estimation of treatment effects in observational data is prone to unmeasured confounding which causes bias. There has been limited research into such biases in a competing risks framework.

**Methods:**

We designed simulations to examine bias in the estimated treatment effect under Cox and Fine & Gray models with unmeasured confounding present. We varied the strength of the unmeasured confounding (i.e. the unmeasured variable’s effect on the probability of treatment and both outcome events) in different scenarios.

**Results:**

In both the Cox and Fine & Gray models, correlation between the unmeasured confounder and the probability of treatment created biases in the same direction (upward/downward) as the effect of the unmeasured confounder on the event-of-interest. The association between correlation and bias is reversed if the unmeasured confounder affects the competing event. These effects are reversed for the bias on the treatment effect of the competing event and are amplified when there are uneven treatment arms.

**Conclusion:**

The effect of unmeasured confounding on an event-of-interest or a competing event should not be overlooked in observational studies as strong correlations can lead to bias in treatment effect estimates and therefore cause inaccurate results to lead to false conclusions. This is true for cause specific perspective, but moreso for a subdistribution perspective. This can have ramifications if real-world treatment decisions rely on conclusions from these biased results. Graphical visualisation to aid in understanding the systems involved and potential confounders/events leading to sensitivity analyses that assumes unmeasured confounders exists should be performed to assess the robustness of results.

**Electronic supplementary material:**

The online version of this article (10.1186/s12874-019-0808-7) contains supplementary material, which is available to authorized users.

## Background

Well-designed observation studies permit researchers to assess treatment effects when randomisation is not feasible. This may be due to cost, suspected non-equipoise treatments or any number of other reasons [1]. While observational studies minimise these issues by being cheaper to run and avoiding randomisation (which, although unknown at the time, may prescribe patients to worse treatments), they are potentially subject to issues such as unmeasured confounding and increased possibility of competing risks (where multiple clinically relevant events occur). Although these issues can arise in any study, Randomised Controlled Trials (RCTs) attempt to mitigate these effects by using randomisation of treatment and strict inclusion/exclusion criteria. However, the estimated treatment effects from RCTs are of potentially limited generalisability, accessibility and implementability [2].

A confounder is a variable that is a common cause of both treatment and outcome. For example, a patient with a high Body Mass Index (BMI) is more likely to be prescribed statins [3], but are also more likely to suffer a cardiovascular event. These treatment decisions can be affected by variables that are not routinely collected (such as childhood socio-economic status or the severity of a comorbidity [4]. Therefore, if these variables are omitted form (or unavailable for) the analysis of treatment effects in observational studies, then they can bias inferences [5]. As well as having a direct effect on the event-of-interest, confounders (along with other covariates) can also have further reaching effects on a patient’s health by changing the chances of having a competing event. Patients who are more likely to have a competing event are less likely to have an event-of-interest, which can affect inferences from studies ignoring the competing event. In the above BMI example, a high BMI can also increase a patient’s likelihood of developing (and thus dying from) cancer [6].

The issue of confounding in observational studies has been researched previously [7–9], where it has been consistently shown that unmeasured confounding is likely to occur within these natural datasets and that there is poor reporting of this, even after the introduction of the The Strengthening the Reporting of Observational Studies in Epidemiology (STROBE) Guidelines [10, 11]. Hence, it is widely recognised that sensitivity analyses are vital within the observational setting [12]. However these previous studies do not extend this work into a competing risk setting, meaning research in this space is lacking [13], particularly where the presence of a competing event can affect the rate of occurrence of the event-of-interest. These issues will commonly occur in elderly and comorbid patients where treatment decisions are more complex. As the elderly population grows, the clinical community needs to understand the optimal way to treat patients with complex conditions; here, causal relationships between treatment and outcome need to account for competing events appropriately.

The most common way of analysing data that contains competing events is using a cause specific perspective, as in the Cox methodology [14], where competing events are considered as censoring events and analysis focuses solely on the event-of-interest. The alternative is to assume a subdistributional perspective, as in the Fine & Gray methodology [15], where patients who have competing events remain in the risk set forever.

The aim of this paper is to study the bias induced by the presence of unmeasured confounding on treatment effect estimates in the competing risks framework. We investigated how unmeasured confounding affects the apparent effect of treatment under the Fine & Gray and the Cox methodologies and how these estimates differ from their true value. To accomplish this, we used simulations to generate synthetic time-to-event-data and then model under both perspectives. Both the Cox and Fine & Gray models provide hazard ratios to describe the effects of a covariate. A binary covariate will represent a treatment and the coefficients found by the model will be the estimate of interest.

## Methods

We considered a simulation scenario in which our population can experience two events; one of which is the event-of-interest (Event 1), the other is a competing event (Event 2). We model a single unmeasured confounding covariate, *U* ~ N (0,1) and a binary treatment indicator, *Z*. We varied how much *U* and *Z* affect the probability distribution of the two events as well as how they are correlated. For example, *Z* could represent whether a patient is prescribed statins, *U* could be their BMI, the event-of-interest could be cardiovascular disease related mortality and a competing event could be cancer-related mortality. We followed best practice for conducting and reporting simulations studies [16].

**Fig. 1:**
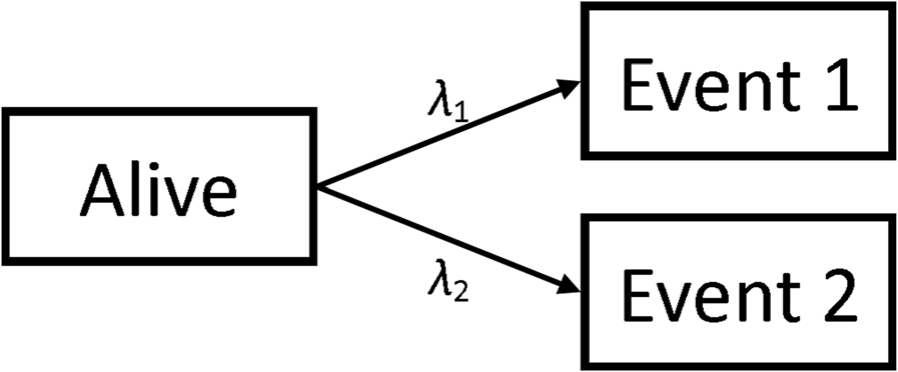
Transition State Diagram showing potential patient pathways

The data-generating mechanism defined two cause-specific hazard functions (one for each event), where the baseline hazard for event 1 was *k* times that of event 2, see Fig. 1. We assumed a baseline hazard that was either constant (exponential distributed failure times), linearly increasing (Weibull distributed failure times) or biologically plausible [17]. The hazards used were thus:

$$ {\lambda}_1\left(t|U,Z\right)=k{e}^{\beta_1U+{\gamma}_1Z}{\lambda}_0(t),\kern0.5em {\lambda}_2\left(t|U,Z\right)={e}^{\beta_2U+{\gamma}_2Z}{\lambda}_0(t) $$

$$ {\lambda}_0(t)=\left\{\begin{array}{cc}1& \mathrm{Exponential}\\ {}2t& \mathrm{Weibull}\\ {}\exp \left(-18+7.3t-11.5{t}^{0.5}\log (t)+9.5{t}^{0.5}\right)& \mathrm{Plausible}\end{array}\right. $$

In the above equations, *β* and *γ* are the effects of the confounding covariate and the treatment effect respectively with the subscripts representing which event they are affecting. These two hazard functions entirely describe how a population will behave [18].

**Fig. 2:**
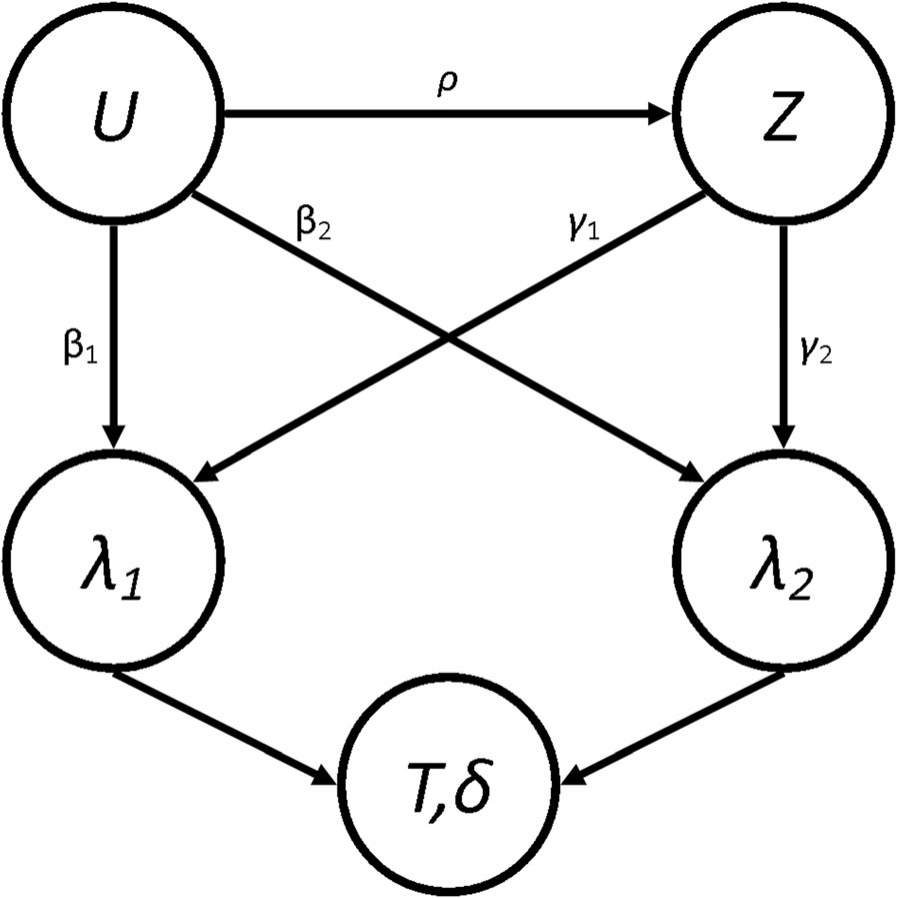
Directed Acyclic Graph showing the relationship between some of the parameters

We simulated populations of 10,000 patients to ensure small confidence intervals around our treatment effect estimates in each simulation. Each simulated population had a distinct value for *β* and *γ*. In order to simulate the confounding of *U* and *Z*, we generated these values such that Corr(*U*,*Z*) = *ρ* and P(*Z* = 1) = *π* [19]. Population end times and type of event were generated using the relevant hazard functions. The full process for the simulations can be found in Additional file 1. Due to the methods used to generate the populations, the possible values for *ρ* are bounded by the choice of π such that when *π* = 0.5, |*ρ*| ≤ 0.797 and when *π* = 0.1 (or 0.9), |*ρ*| ≤ 0.57. The relationship between the parameters can be seen in the Directed Acyclic Graph (DAG) shown in Fig. 2, where *T* is the event time and *δ* is the event type indicator (1 for event-of-interest and 2 for competing event).

From this, we also explicitly calculated what we would expect the true subdistribution treatment effects, *Γ*_*1*_ and *Γ*_*2*_, to be in these conditions [20] (See Additional file 2). It’s worth noting that the values of *Γ* will depend on the current value of *ρ* since they are calculated using the expected distribution of end-times. However, it has been shown [18, 21] that, due to the relationship between the Cause-Specific Hazard (CSH) and the Subdistribution Hazard (SH), only one proportional hazards assumption can be true. Therefore the “true” values of the *Γ* will be misspecified and represent a least false parameter (which itself is an estimate of the time-dependent truth) [20].

We used the simulated data to estimate the treatment effects under the Cox and Fine & Gray regression methods. We specify that *U* is unmeasured and so it wasn’t included in the analysis models. As discussed earlier, the Cox model defines the risk set at time *t* to be all patients who have not had *any* event by time *t*, whereas the Fine & Gray defines it to be those who have not had the event-of-interest (or competing event) by time *t*.

For our models, for the events, *i* = {1,2}, we therefore defined the CSH function estimate, *λ̂*_*i,*_ and the SH function estimate, *ĥ*_*i*_, to be

$$ \hat{\lambda_i}\left(t|Z\right)=\hat{\lambda_{i0}}(t){e}^{\hat{\gamma_i}Z}\kern0.5em \hat{h_i}\left(t|Z\right)=\hat{h_{i0}}(t){e}^{\hat{\varGamma_i}Z} $$

Where *λ̂*_*i0*_ and *ĥ*_*i0*_ are the baseline hazard and baseline subdistribution hazard function estimates for the entire population (i.e. no stratification), and *γ̂*_*i*_ and *Γ̂*_*i*_ are the estimated treatment effects. From these estimates, we also extracted the estimate of the subdistribution treatment effect in a hypothetical RCT, where *ρ* = 0 and π = 0.5 to give *Γ̂*_*10*_ and *Γ̂*_*20*_. To investigate how the correlation between *U* and *Z* affects the treatment effect estimate, we compared the explicitly prescribed or calculated values with the simulated estimates. Three performance measures for both events, along with appropriate 95% confidence intervals, were calculated for each set of parameters:

- *θ*_*RCT,i*_ = E [*Γ̂*_*i*_ *- Γ̂*_*i0*_] ~ The average difference between the SH treatment effect estimate from an idealised, hypothetical RCT situation.
- *θ*_*Exp,i*_ = E [*Γ̂*_*i*_ *– Γ*_*i*_] ~ The average bias of the SH treatment effect estimate from the explicitly calculated value.
- *θ*_*CSH,i*_ = E [*γ̂*_*i*_ *-γ*_*i*_] ~ The average bias of the CSH treatment effect estimate from the predefined treatment effect.

As mentioned above, the value of *Γ* will depend on the current value of *ρ* and so the estimation of the explicit bias will be a measure of the total bias induced on our estimate of the subdistribution treatment effect in those specific set of parameters. We also evaluate the bias compared to an idealised RCT to see how much of this bias could be mitigated if we were to perform an RCT to assess the effectiveness of the hypothetical treatment. Finally, we found the explicit bias in the cause specific treatment effect to again see the total bias applied to this measure. We did not compared the CSH bias to an idealised RCT as we believed that this could easily be inferred from the CSH explicit results, whereas this information wouldn’t be as obvious in the SH treatment effect due to the existence of a relationship between *Γ* and *ρ*.

Eight Scenarios were simulated based on real-world situations. In each scenario, *ρ* varied across 5 different values ranging from 0 to their maximum possible value (0.797 for all Scenarios apart from Scenario 5, where it is 0.57, due to the bounds imposed by the values of *π*). One other parameter (different for different scenarios) varied across 3 different values, and all other parameters were fixed as detailed in Table 1. Each simulation was run 100 times and the performance measures were each pooled to provide small confidence intervals. This gives a total of 1,500 simulations for each of the 8 scenarios. Descriptions of the different scenarios are given below:

1. No Effect. To investigate whether treatment with no true effect (*γ*_*1*_ = *γ*_*2*_ = 0) can have an “artificial” treatment effect induced on them in the analysis models through the confounding effect on the event-of-interest. *β*_*1*_ varied between − 1, 0 and 1.
2. Positive Effect. To investigate whether treatment effects can be reversed when the treatment is beneficial for both the event-of-interest and the competing event (*γ*_*1*_ = *γ*_*2*_ = − 1). *β*_*1*_ varied between − 1, 0 and 1.
3. Differential Effect. To investigate how treatment effect estimates react when the effect is different for the event-of-interest (*γ*_*1*_ = − 1) and the competing event (*γ*_*2*_ = 1). *β*_*1*_ varied between − 1, 0 and 1.
4. Competing Confounder. To investigate whether treatments with no true effect (*γ*_*1*_ = *γ*_*2*_ = 0) can have an “artificial” treatment effect induced on them by the effect of a confounded variable on the competing event only (β_*1*_ = 0). *β*_*2*_ varied between − 1, 0 and 1.
5. Uneven Arms. To investigate how having uneven arms on a treatment in the population can have an effect on the treatment effect estimate (*γ*_*1*_ = − 1, *γ*_*2*_ = 0). π varied between ^1^/_10_, ½ and ^9^/_10_.
6. Uneven Events. To investigate how events with different frequencies can induce a bias on the treatment effect, despite no treatment effect being present (*γ*_*1*_ = *γ*_*2*_ = 0). *k* varied between ½, 1 and 2.
7. Weibull Distribution. To investigate whether a linearly increasing baseline hazard function affects the results found in Scenario 1. *β*_*1*_ varied between − 1, 0 and 1.
8. Plausible Distribution. To investigate whether a biologically plausible baseline hazard function affects the results found in Scenario 1. *β*_*1*_ varied between − 1, 0 and 1.

**Table 1:** Details of the parameters for each Scenario

Sc	*ρ*					Baseline	*γ*_*1*_	*γ*_*2*_	*β*_*1*_			*β*_*2*_			*π*			*k*		
1	0	0.20	0.40	0.60	0.80	Constant	0	0	−1	0	1	0			^1^/_2_			1		
2	0	0.20	0.40	0.60	0.80	Constant	−1	−1	− 1	0	1	0			^1^/_2_			1		
3	0	0.20	0.40	0.60	0.80	Constant	−1	1	−1	0	1	0			^1^/_2_			1		
4	0	0.20	0.40	0.60	0.80	Constant	0	0	0			−1	0	1	^1^/_2_			1		
5	0	0.14	0.29	0.42	0.57	Constant	0	0	1			0			^1^/_10_	^1^/_2_	^9^/_10_	1		
6	0	0.20	0.40	0.60	0.80	Constant	0	0	1			0			^1^/_2_			^1^/_2_	1	2
7	0	0.20	0.40	0.60	0.80	Weibull	0	0	−1	0	1	0			^1^/_2_			1		
8	0	0.20	0.40	0.60	0.80	Plausible	0	0	−1	0	1	0			^1^/_2_			1		

## Results

**Fig. 3:**
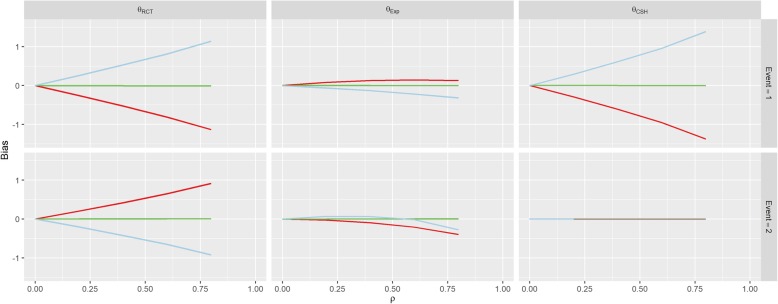
Results from Scenario 1. Legend

The first row of Fig. 3 shows the results for Scenario 1 (No Effect). When *β*_*1*_ = *β*_*2*_ = 0 (the green line), correlation between *U* and *Z* doesn’t imbue any bias on the treatment effect estimate for either event under any of the three measures, since all of the subdistribution treatment effects (estimated, calculated and hypothetical RCT) are approximately zero. When *β*_*1*_ > 0, there is a strong positive association between correlation (*ρ*) and the RCT and CSH biases for the event-of-interest and a negative association for the RCT bias for the competing event. Similarly, these associations are reversed when *β*_*1*_ < 0.

**Fig. 4:**
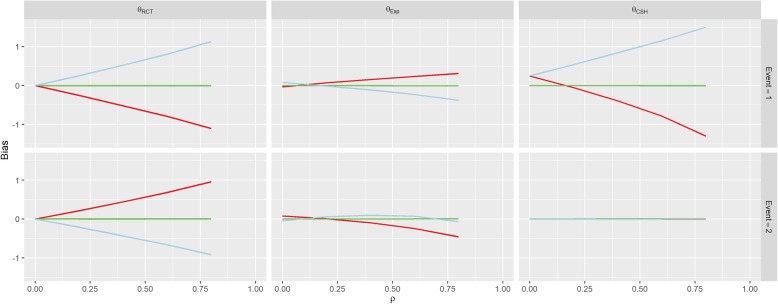
Results from Scenario 2 Legend

**Fig. 5:**
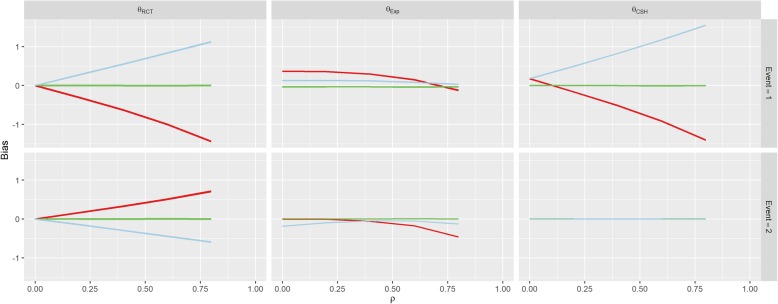
Results from Scenario 3 Legend

There was no effect on *θ*_*CSH*_ for the competing event in this Scenario regardless of *ρ* or *β*_*1*_. These results are similar to those found in Scenario 2 (Positive Effect) and Scenario 3 (Negative Effect) shown in Figs. 4 and 5. However, in both of these Scenarios, there is an overall positive shift in *θ*_*CSH*_ when *β*_*1*_ ≠ 0.

The magnitude of *θ*_*Exp*_ is greatly reduced and is the reverse of the other associations when *β*_*1*_ ≠ 0 in Scenario 1 for the event-of-interest and when *β*_*1*_ > 0 it stays extremely small for low values of *ρ*, and becomes negative for large *ρ* for the competing event. In Scenario 2, *θ*_*Exp*_ behaves similarly to Scenario 1 for both events when *β*_*1*_ < 0 and the event-of-interest, but for the competing event, when *β*_*1*_ > 0, the *θ*_*Exp*_ is much tighter to 0. The competing event data for *θ*_*Exp*_ in Scenario 3 is similar to Scenario 2 with *β*_*1*_ > 0 shifted downwards, but the event-of-interest has a near constant level of bias regardless of *ρ*, apart from in the case when *β*_*1*_ < 0, the bias switches direction.

**Fig. 6:**
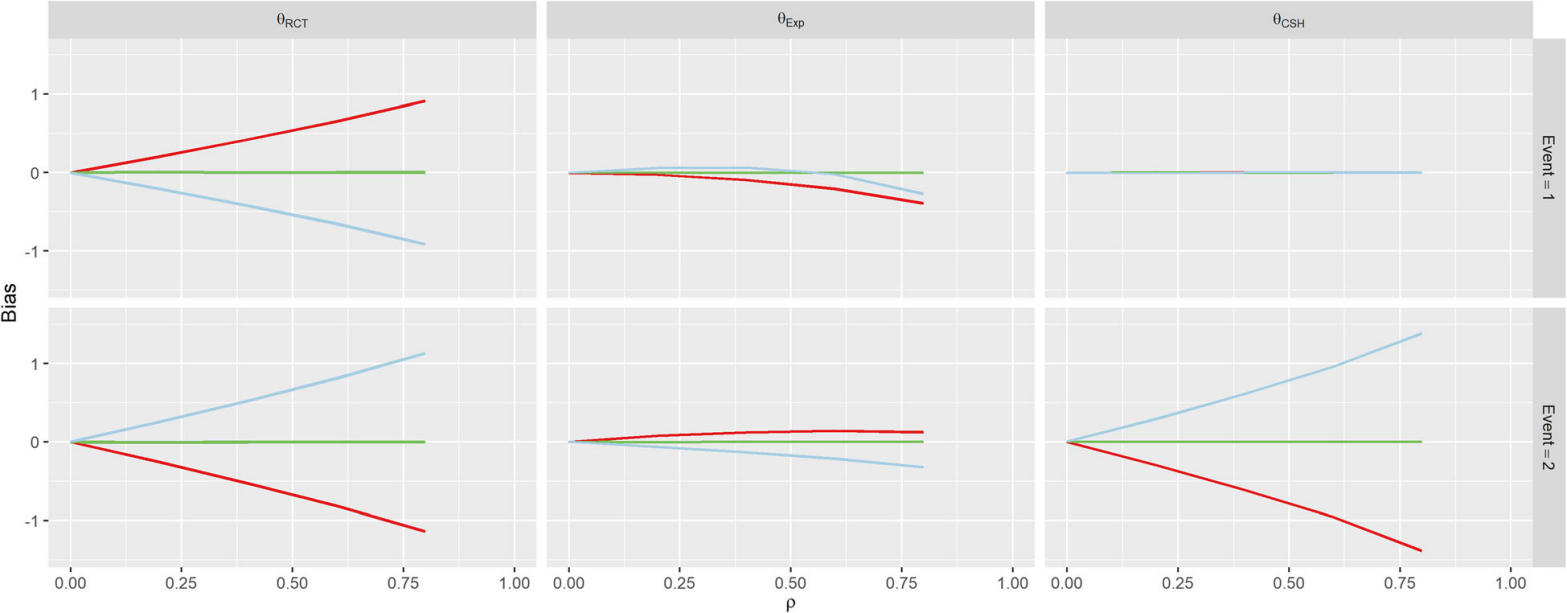
Results from Scenario 4 Legend

**Fig. 7:**
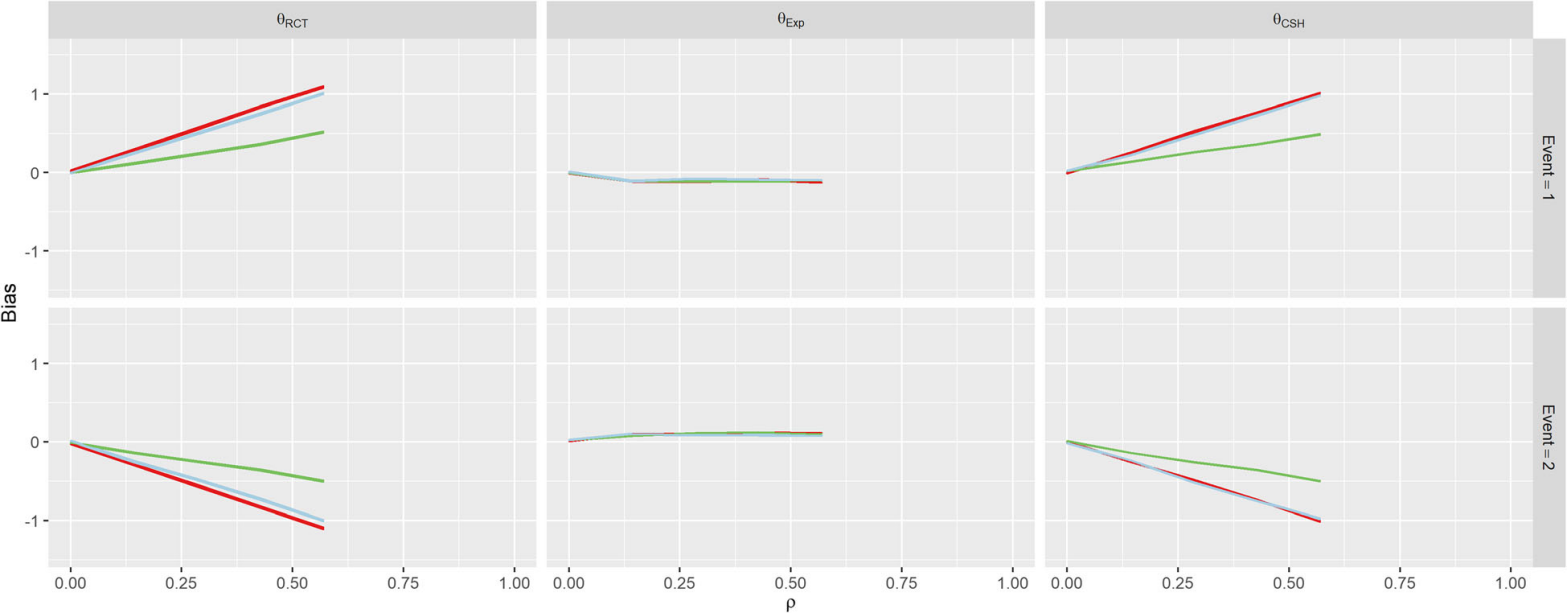
Results from Scenario 5 Legend

In Scenario 4 (Competing Confounder), as would be expected, the results for the event-of-interest and the results for the competing event are swapped from those of Scenario 1 as shown in Fig. 6. Scenario 5 (Uneven Arms) portrays a bias similar to Scenario 1 where *β*_*1*_ = 1, however, the magnitude of the RCT and CSH bias is increased when *π* ≠ 0.5 as shown in Fig. 7.

**Fig. 8:**
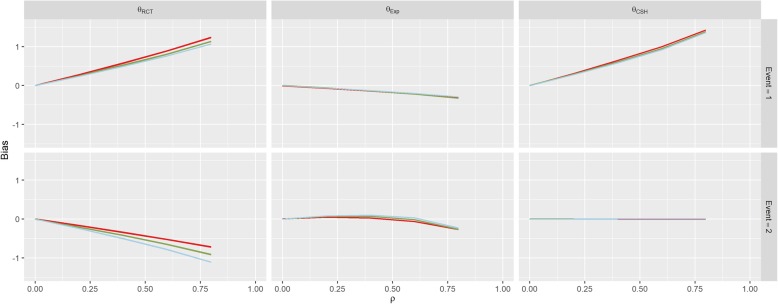
Results from Scenario 6 Legend

**Fig. 9:**
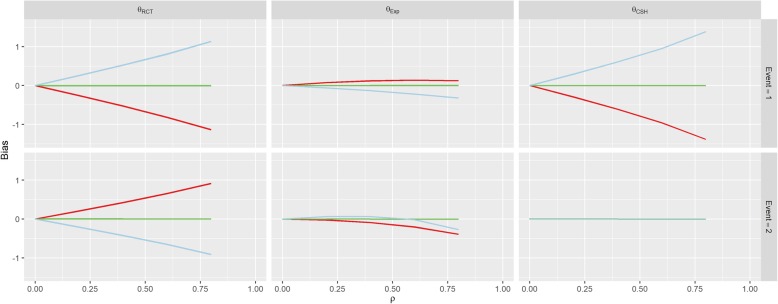
Results from Scenario 7 Legend

**Fig. 10:**
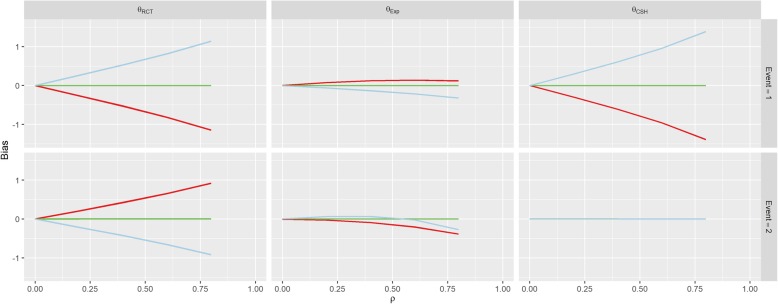
Results from Scenario 8 Legend

The parameters for Scenario 6 (Uneven Events) were similar to the parameters for Scenario 1 (No Effect), when *β*_*1*_ = 1. This also reflects in the results in Fig. 8 which look similar to the results for this set of parameters in Scenario 1. This bias is largely unaffected by the value of *k*. The results of Scenario 7 (Weibull Distribution) and Scenario 8 (Plausible Distribution) were nearly identical to those of Scenario 1 as shown in Figs. 9 and 10.

As per our original hypotheses, Scenario 1 demonstrated that it is possible to induce a treatment effect when one isn’t present through confounding effects on all biases, apart from the competing event CSH. In Scenario 2, with high enough correlation, the CSH event-of-interest bias could be greater than 1, meaning that the raw CSH treatment effect was close to 0, despite an actual treatment effect of − 1, similarly large positive biases in the SH imply a treatment with no benefit and/or detrimental effect, despite the true treatment being beneficial for both events. This finding is similar for Scenario 3 with large biases changing the direction of the treatment effect (beneficial vs detrimental).

Scenario 4 demonstrated that even without a treatment effect and with no confounding effect on the event-of-interest, a treatment effect can be induced on the SH methodology, which can imply a beneficial/detrimental treatment, depending on whether the confounder was detrimental/beneficial. Fortunately, it does not induce an effect on the CSH treatment effect for the event-of-interest.

Scenarios 5 and 6 investigated other population level effects; differences in the size of the treatment arms and differences in the magnitude of the hazards of the events. Scenario 5 demonstrated that having uneven treatment arms can exacerbate the bias induced on both the *θ*_*RCT*_ and *θ*_*CSH*_ for both events and Scenario 6 showed that the different baseline hazards had little effect on the levels of bias in the results. This finding was supported by the additional findings of Scenarios 7 and 8, which showed that the underlying hazard functions did not affect the treatment effect biases compared to a constant hazard.

## Discussion

This is the first paper to investigate the issue of unmeasured confounding on a treatment effect in a competing risks scenario. Herein, we have demonstrated that regardless of the actual effect of a treatment on a population that is susceptible to competing risks, bias can be induced by the presence of unmeasured confounding. This bias is largely determined by the strength of the confounding relationship with the treatment decision and size of confounding effect on both the event-of-interest and any competing events. This effect is present regardless of any difference in event rates between the events being investigated and is also exacerbated by misbalances in the number of patients who received treatment and the number of patients who did not.

Our study has shown how different the case would be if a similar population (without inclusion/exclusion criteria) were put through an RCT and how the correlation between an unmeasured confounder and the treatment is removed, as would be the case in a pragmatic RCT. By combining the biases from an RCT and the explicitly calculated treatment effect, we can also use these results to infer how much of the bias found here is from omitted variable bias [22] and how much is explicitly due to the correlation between the covariates. Omitted variable bias occurs when a missing covariate has an effect on the outcome, but is not correlated with the treatment (and so is not a true confounder). It can occur even if the omitted variable is initially evenly distributed between the two treatment arms because, as patients on one arm have events earlier than the other, the distributions of the omitted variable drift apart. This makes up some of the bias caused by unmeasured confounding, but not all of it. For example, in Scenario 3 (Differential Effect), the treatment lowered the hazard of the event-of-interest, but increased the hazard of the competing event; with a median level of correlation (*ρ* = 0.4), the event-of-interest bias from the RCT when there is a negative confounding effect (*β*_*1*_ < 0) is − 0.628 and the bias from the explicit estimate is 0.295 and therefore, the amount of bias due purely to the correlation between the unmeasured confounder and the treatment is actually − 0.923. In this instance, some of the omitted variable bias is actually mitigating the bias from the correlation; if we have two biasing effects that can potentially cancel each other out, we could encounter a Type III error [23] which is very difficult to prove and can cause huge problems for reproducibility (if you eliminate a single source of bias, your results will be farther from the truth).

Our simulations indicate that a higher (lower) value of *β*_*1*_ and a lower (higher) value of *β*_*2*_ will produce a higher (lower) bias in the event-of-interest. These two biasing effects could cancel out to produce a situation similar to above. In our scenarios, we saw that, even when a treatment has no effect on the event-of-interest or a competing event (i.e. the treatment is a placebo), both a cause specific treatment effect and a subdistribution treatment effect can be found. This also implies that the biasing effect of unmeasured confounders (both omitted variable and correlation bias) can result in researchers reaching incorrect conclusions about how a treatment affects a population in multiple ways. We could have a treatment that is beneficial for the prevention of both types of event, but due to the effects of an unmeasured confounder, it could be found to have a detrimental effect (for one or both) on patients from a subdistribution perspective.

Our investigation augments Lin et al’s study into unmeasured confounding in a Cox model [5] by extending their conclusion (that bias is in the same direction as the confounder’s effect and dependent on its strength) into a competing risks framework (i.e. by considering the Fine & Gray model as well) and demonstrating that this effect is reversed when there is confounding with the competing event. Lin et al. [5] also highlight the problems of omitted variable bias, which comes from further misspecification of the model; this finding was observed in our results as described above for Scenario 3.

The results from Scenario 7 (Weibull Distribution) and Scenario 8 (Plausible Distribution) are almost identical to those of Scenario 1 (No Effect) which implies that, by assuming both hazard functions in question are the same, we can assume they are both constant for simplicity. Since both the Cox and Fine & Gray models are ambiguous to underlying hazard functions and treatment effects are estimated without consideration for the baseline hazard function, it makes intuitive sense that the results would be identical regardless of what underlying functions were used to generate our data. This makes calculation of the explicit subdistribution treatment effect much simpler for future researchers.

Thompson et al. used the paradox that smoking reduces melanoma risk to motivate simulations similar to ours, which demonstrated how the exclusion of competing risks, when assessing confounding, can lead to unintuitive, mis-specified and possibly dangerous conclusions [24]. They hypothesised that the association found elsewhere [25] may be caused by bias due to ignoring competing events and used Monte Carlo simulations to provide examples of scenarios where these results would be possible. They demonstrated how a competing event could cause incorrect conclusions when that competing event is ignored – a conclusion we also confirm through the existence of bias induced on the Cox modelled treatment effect even with no correlation between the unmeasured confounder and treatment (i.e. *θ*_*CSH,1*_ ≠ 0 when *ρ* = 0 in Scenarios 2 & 3). Thompson’s team began with a situation where there may be a bias due to a competing event and reverse-engineered a scenario to find the potential sources of bias, whereas our study explored different scenarios and investigated the biased results they potentially produced.

Groenwold et al. [26] proposed methods to perform simulations to evaluate how much unmeasured confounding would be necessary for a true effect to be null given that an effect has been found in the data. Their methods can easily be applied to any metric in clinical studies (such as the different hazard ratios estimated here). Currently, epidemiologists will instigate methods such as DAGs, see Fig. 2, to visualise where unmeasured confounding may be a problem in analysis [27] and statisticians who deal with such models will use transition diagrams, see Fig. 1, to visualise potential patient pathways [28]. Using these two visualisation techniques in parallel will allow researchers to anticipate these issues, successfully plan to combat them (through changes to protocol or sensitivity analysis, etc. …) and/or implement simulations to seek hidden sources of bias (using the methods of Groenwold [26] and Thompson [24]) or to adjust their findings by assuming biases similar to those demonstrated in our paper exist in their work.

The work presented here could be extended to include more complicated designs such as more competing events, more covariates and differing hazard functions. However, the intention of this paper was to provide a simple dissection of specific scenarios that allow for generalisation to clinical work. The main limitation of this work, to use of the same hazard functions for both events in each of our scenarios, was a pragmatic decision made to reduce computation time. The next largest limitation was the lack of censoring events, and was chosen to simplify interpretation of the model. This situation is unlikely to happen in the real world. However, since both the Cox and the Fine & Gray modelling techniques are robust to any underlying baseline hazard and independent censoring of patients [14, 15, 29], these simplifications should not have had a detrimental effect on the bias estimates given in this paper. This perspective on censoring is similar to the view of Lesko et al. [30] in that censoring would provide less clarity of the presented results.

## Conclusion

This paper has demonstrated that unmeasured confounding in observational studies can have an effect on the accuracy of outcomes for both a Cox and a Fine & Gray model. We have added to the literature by incorporating the effect of confounding on a competing event as well as on the event-of-interest simultaneously. The effect of confounding is present and reversed compared to that of confounding on the event-of-interest. This makes intuitive sense as a negative effect on a competing event has a similar effect at the population level as a positive effect on the event-of-interest (and vice versa). This should not be overlooked, even when dealing with populations where the potential for competing events is much smaller than potential for the event-of-interest and is especially true when the two arms of a study are unequal. Therefore, we recommend that research with the potential to suffer from these issues be accompanied by sensitivity analyses investigating potential unmeasured confounding using established epidemiological techniques applied to any competing events as well as the event-of-interest. In short, unmeasured variables can cause problems with research, but by being knowledgeable about what we don’t know, we can make inferences despite this missing data.

## Additional files

Additional file 1: Details of the simulation process. (DOCX 20 kb)

Additional file 2: Details of the mathematics behind the estimation of the “true” subdistribution hazard treatment effect. (DOCX 28 kb)

## Data Availability

The datasets used and/or analysed during the current study are available from the corresponding author on reasonable request.

## Abbreviations

BMI: Body Mass Index
CSH: Cause-Specific Hazard
DAG: Directed Acyclic Graph
RCT: Randomised Controlled Trial
SH: Subdistributional Hazard
