# Supplementary material for: How unmeasured confounding in a competing risks setting can affect treatment effect estimates in observational studies
Source: BMC Med Res Methodol. 2019 Jul 31;19:166. doi: 10.1186/s12874-019-0808-7 (PMC6668192; doi:10.1186/s12874-019-0808-7)
Supplement: Supplementary file 1 — Details of the simulation process. (DOCX 20 kb) [file 12874_2019_808_MOESM1_ESM.docx]

# Appendix A

The populations used in these simulations are generated such that *Corr(U,Z) = ρ* and *P(Z=1) = π*. These baseline populations are then acted on by the event-of-interest and competing event hazard functions. These are combined into the cumulative hazard function. These are defined as follows:

$$\begin{matrix} \lambda_{1}\left( t | U,Z \right)=ke^{\beta_{1}U+\gamma_{1}Z}\lambda_{0}\left( t \right), & \lambda_{2}\left( t | U,Z \right)=e^{\beta_{2}U+\gamma_{2}Z}\lambda_{0}(t) \end{matrix}$$

$$\Lambda\left( t | U,Z \right)=\int_{0}^{t} \lambda_{1}\left( s | U,Z \right)+\lambda_{2}\left( s | U,Z \right)\text{ d}s$$

Let *φ* and *Φ* be the pdf and cdf for the standard Normal Distribution respectively and *I* be the identity function. The process for generating the covariates and outcome data for a patient is given in Table A. 1.

| Step | Variable | Calculation | Description |
| --- | --- | --- | --- |
| 1 | *x_0_* | $x_{0}=\Phi^{-1}(\pi)$ | This is the value of *x ~ N(0,1)* such that *P(x < x_0_) = π* |
| 2 | *r* | $r=\frac{\rho\sqrt{\pi\left( 1-\pi\right)}}{\varphi\left( x_{0} \right)}$ | This is used to ensure *Corr(U,Z) = ρ* |
| 3 | *Y_1_* | $Y_{1i}\sim N(0,1)$ | Generate 10,000 random normal numbers |
| 4 | *Y_2_* | $Y_{2i}\sim N(0,1)$ | Generate 10,000 random normal numbers |
| 5 | *U* | $U_{i}=Y_{1i}r+Y_{2i}\sqrt{1-r^{2}}$ | This defines *U~N(0,1)* such that *Corr(U,Y_1_) = r* |
| 6 | *Z* | $Z_{i}=I(\Phi\left( Y_{1i} \right)<\pi)$ | This defines *Z* such that *P(Z=1)=π* |
| 7 | *λ_1_(t)* | $\lambda_{1i}\left( t \right)=\lambda_{1}(t\vert U_{i},Z_{i})$ | Define an array of hazard functions |
| 8 | *λ_2_(t)* | $\lambda_{2i}\left( t \right)=\lambda_{2}(t\vert U_{i},Z_{i})$ | Define an array of hazard functions |
| 9 | *Λ(t)* | $\Lambda_{i}\left( t \right)=\Lambda(t\vert U_{i},Z_{i})$ | Define an array of cumulative hazard functions |
| 10 | *S(t)* | $S_{i}\left( t \right)=\exp(-\Lambda_{i}\left( t \right))$ | Define an array of Survival functions |
| 11 | *V_1_* | $V_{1i}\sim Unif(0,1)$ | Generate 10,000 random uniform numbers |
| 12 | *V_2_* | $V_{2i}\sim Unif(0,1)$ | Generate 10,000 random uniform numbers |
| 13 | *T* | $T_{i}=S_{i}^{-1}(V_{1i})$ | Solve (numerically or explicitly depending on *λ_0_*) |
| 14 | *δ* | $\delta_{i}=1+I\left( V_{2}>\frac{\left( \lambda_{1i}\left( T_{i} \right) \right)}{\left( \lambda_{1i}\left( T_{i} \right)+\lambda_{2i}\left( T_{i} \right) \right)} \right)$ | Generates which event occurs |

Table A. 1 Table showing the steps taken to generate each simulated population

Due to the square root in Step 5, we restrain |*r*| < 1. This coupled with the definition of *r* in Step 2 means that the values of *ρ* and *π* are limited by each other such that:

$$\left| \frac{\rho\sqrt{\pi\left( 1-\pi\right)}}{\phi\left( x_{0} \right)} \right|<1$$

Therefore, we have bounds on *ρ* dependent on *π*:

$$\left| \rho\right|<\frac{\phi\left( x_{0} \right)}{\sqrt{\pi\left( 1-\pi\right)}}$$

Since *x_0_* is dependent on *π*, *φ(x_0_)* is also dependent on *π* and can be approximated by

$$\phi\left( x_{0} \right)\approx\frac{8}{5}\pi(1-\pi)$$

And thus the bound on ρ can be approximated by:

$$\left| \rho\right|<\frac{8}{5}\sqrt{\pi\left( 1-\pi\right)}$$

This provides us with the bounds discussed in the main text.
