# Supplementary material for: How unmeasured confounding in a competing risks setting can affect treatment effect estimates in observational studies
Source: BMC Med Res Methodol. 2019 Jul 31;19:166. doi: 10.1186/s12874-019-0808-7 (PMC6668192; doi:10.1186/s12874-019-0808-7)
Supplement: Supplementary file 2 — Details of the mathematics behind the estimation of the “true” subdistribution hazard treatment effect. (DOCX 28 kb) [file 12874_2019_808_MOESM2_ESM.docx]

# Appendix B

Due to the relationship between the cause specific hazard functions and the subdistribution hazard functions they cannot both satisfy the proportional hazards assumption. We have defined CSH functions to be proportional and so the SH functions are not. In order to find the “true” SH treatment effect for the event-of-interest, we have to find the least false parameter [20]. To do this, we must solve the following equation for *b(Γ_1_) = 0*:

$$b\left( \Gamma_{1} \right)=\int_{0}^{\infty} \frac{\left( 1-F_{1}\left( t | Z=0 \right) \right)f_{1}\left( t | Z=1 \right)-e^{\Gamma_{1}}\left( 1-F_{1}\left( t | Z=1 \right) \right)f_{1}(t|Z=0)}{\left( 1-\pi\right)\left( 1-F_{1}\left( t | Z=0 \right) \right)+\pi e^{\Gamma_{1}}(1-F_{1}\left( t | Z=1 \right))}\text{ d}t$$

where *f_1_* and *F_1_* are the pdf and cdf of the subdistribution for the event-of-interest. These are defined as

$$f_{1}\left( t | Z \right)=\lambda_{1}\left( t | Z \right)S\left( t | Z \right)$$

$$F_{1}\left( t | Z \right)=\int_{0}^{t} f_{1}\left( u | Z \right)\text{ d}u$$

In populations where only a single event occurs, these are the same as the pdf and cdf of the distribution for the event. We have previously defined our hazard functions as (with *k_j_* = *k* if *j* = 1 and 1 otherwise)

$$\lambda_{j}\left( t | U,Z \right)={k_{j}e}^{\beta_{j}U+\gamma_{j}Z}\lambda_{0}(t)$$

and so to find λj(t|Z), we need to eliminate *U* from our equations by finding the expectation of *U* given *Z*.

$$E\left[ e^{\beta U} | Z=0 \right]=E\left[ e^{\beta U} | Y_{1}<x_{0} \right]$$

$$=E\left[ e^{\beta\left( rY_{1}+\sqrt{1-r^{2}}Y_{2} \right)} | Y_{1}<x_{0} \right]$$

$$=E\left[ e^{Y_{2}} \right]^{\beta\sqrt{1-r^{2}}}\times E\left[ e^{Y_{1}} | Y_{1}<x_{0} \right]^{\beta r}$$

$$=\left( e^{\frac{1}{2}} \right)^{\beta\sqrt{1-r^{2}}}\times\left( \int_{-\infty}^{x_{0}} e^{y}\left( \frac{\phi\left( y \right)}{\Phi\left( x_{0} \right)} \right)\text{ d}y \right)^{\beta r}$$

$$=\frac{e^{\frac{\beta\left( \sqrt{1-r^{2}} \right)}{2}}}{1-\pi}\times\left( \int_{-\infty}^{x_{0}} e^{y}\phi\left( y \right)\text{ d}y \right)^{\beta r}$$

$$=\frac{e^{\frac{\beta\left( \sqrt{1-r^{2}} \right)}{2}}}{1-\pi}\times e^{\frac{\beta r}{2}}\left( \int_{-\infty}^{x_{0}} \frac{1}{\sqrt{\tau}}e^{-\frac{1}{2}\left( y-1 \right)^{2}}\text{ d}y \right)^{\beta r}$$

$$=\frac{e^{\frac{\beta}{2}\left( r+\sqrt{1-r^{2}} \right)}}{1-\pi}\times\left( \int_{-\infty}^{x_{0}-1} \frac{1}{\sqrt{\tau}}e^{-\frac{1}{2}w^{2}}\text{ d}w \right)^{\beta r}$$

$$E\left[ e^{\beta U} | Z=0 \right]=\frac{e^{\frac{\beta}{2}\left( r+\sqrt{1-r^{2}} \right)}\left( \Phi\left( x_{0}-1 \right) \right)^{\beta r}}{1-\pi}$$

$$s average value that n$$

Walking through each step:

1. We defined *Z* from *Y_1_* and so we can substitute *Z=0* with *Y_1_* < *x_0_* (See Appendix A).
2. We defined *U* based on *Y_1_* and *Y_2_* so we can substitute this formula in (See Appendix A).
3. The expectation of a product is product of the expectations so this can be split. Similarly, powers are taken out of the expectation. *Y_1_* and *Y_2_* are independent so is dropped from the left-hand expectation.
4. First expectation follows since *Y_2_ ~ N(0,1)*. The second is the expectation of a truncated Normal distribution.
5. By definition, *Φ(x_0_) = 1-π*, so we can replace and bring it out of the integral.
6. Substituting the formula for *φ(y)* (Normal pdf). Note that *τ* is used rather than *2π* to avoid notation confusion. The powers of *e* are combined and simplified (using completing the square) and the constant is taken out of the integral
7. Substituting *w = y-1* inside the integral, and not forgetting to change the limit.
8. The formula under the integration is again the Normal pdf, so we evaluate the Normal cdf.

Similarly, for Z=1, we swap the less than sign in the first line and evaluate the integral on a different range:

$$E\left[ e^{\beta U} | Z=1 \right]=E\left[ e^{\beta U} | Y_{1}>x_{0} \right]$$

$$=E\left[ e^{\beta\left( rY_{1}+\sqrt{1-r^{2}}Y_{2} \right)} | Y_{1}>x_{0} \right]$$

$$=E\left[ e^{Y_{2}} \right]^{\beta\sqrt{1-r^{2}}}\times E\left[ e^{Y_{1}} | Y_{1}>x_{0} \right]^{\beta r}$$

$$=\left( e^{\frac{1}{2}} \right)^{\beta\sqrt{1-r^{2}}}\times\left( \int_{x_{0}}^{\infty} e^{y}\left( \frac{\phi\left( y \right)}{1-\Phi\left( x_{0} \right)} \right)\text{ d}y \right)^{\beta r}$$

$$=\frac{e^{\frac{\beta\left( \sqrt{1-r^{2}} \right)}{2}}}{\pi}\times\left( \int_{x_{0}}^{\infty} e^{y}\phi\left( y \right)\text{ d}y \right)^{\beta r}$$

$$=\frac{e^{\frac{\beta\left( \sqrt{1-r^{2}} \right)}{2}}}{\pi}\times e^{\frac{\beta r}{2}}\left( \int_{x_{0}}^{\infty} \frac{1}{\sqrt{\tau}}e^{-\frac{1}{2}\left( y-1 \right)^{2}}\text{ d}y \right)^{\beta r}$$

$$=\frac{e^{\frac{\beta}{2}\left( r+\sqrt{1-r^{2}} \right)}}{\pi}\times\left( \int_{x_{0}-1}^{\infty} \frac{1}{\sqrt{\tau}}e^{-\frac{1}{2}w^{2}}\text{ d}w \right)^{\beta r}$$

$$E\left[ e^{\beta U} | Z=0 \right]=\frac{e^{\frac{\beta}{2}\left( r+\sqrt{1-r^{2}} \right)}\left( 1-\Phi\left( x_{0}-1 \right) \right)^{\beta r}}{\pi}$$

By defining *π_1_* = *1-Φ(x_0_-1)*, these can be combined to be:

$$E\left[ e^{\beta U} | Z=z \right]=\left( e^{\frac{\beta}{2}\left( r+\sqrt{1-r^{2}} \right)} \right)\left( \frac{\left( z\pi_{1}+\left( 1-z \right)\left( 1-\pi_{1} \right) \right)^{\beta r}}{z\pi+\left( 1-z \right)\left( 1-\pi\right)} \right)$$

Substituting this into the equation for *λ_j_(t|Z)* gives:

$$\lambda_{j}\left( t | Z \right)=k_{j}{\left( e^{\gamma_{j}Z+\frac{\beta_{j}}{2}\left( r+\sqrt{1-r^{2}} \right)} \right)\left( \frac{\left( Z\pi_{1}+\left( 1-Z \right)\left( 1-\pi_{1} \right) \right)^{\beta r}}{Z\pi+\left( 1-Z \right)\left( 1-\pi\right)} \right)\lambda}_{0}(t)$$

This can then be used to produce a formula for *Λ(t|Z)* and then *S(t|Z)*. From *λ_1_(t|Z)* and *S(t|Z)*, we can find *f_1_(t|Z)* and *F_1_(t|Z)* using the above equations and thus *Γ_1_* can be found. This process can be repeated to find *Γ_2_* by exchanging *f_1_* and *F_1_* for *f_2_* and *F_2_* and evaluating similarly.
